# *Phytophthora inundata*: A New Root Pathogen of Citrus in Europe and the Mediterranean Region

**DOI:** 10.3390/plants14091333

**Published:** 2025-04-28

**Authors:** Cristian Bua, Maria Catena Tambè, Sebastiano Conti Taguali, Mario Riolo, Alessandro Vitale, Antonella Pane, Santa Olga Cacciola

**Affiliations:** Department of Agriculture, Food and Environment, University of Catania, Via S. Sofia 100, 95123 Catania, Italy; cristian.bua@phd.unict.it (C.B.); tambemaria94@gmail.com (M.C.T.); alessandro.vitale@unict.it (A.V.); antonella.pane@unict.it (A.P.); olga.cacciola@unict.it (S.O.C.)

**Keywords:** *Phytophthora* clade 6, root rot, *Phytophthora nicotianae*, multi-gene phylogenetic analysis, multiple infection, complex diseases

## Abstract

Citruses are one of the major fruit crops globally. Among Mediterranean citrus producers, Sicily (southern Italy) is renowned for its high-quality fresh fruit production. Phytophthora diseases are a serious issue for citrus production worldwide and *Phytophthora nicotianae* is a prevalent causal agent of root rot in most citrus growing areas globally and particularly in the Mediterranean region. This study reports the occurrence of *Phytophthora inundata* as a root pathogen of declining mature citrus trees in eastern Sicily in association with *P. nicotianae*. This is the first record of *P. inundata* on citrus in Europe and the Mediterranean region. The species was identified on the basis of a morphology and multi-gene phylogenetic analysis, which included the internal transcribed spacer, β-tubulin and cytochrome c oxidase subunit 1. Pathogenicity tests on citrus saplings showed *P. inundata* was a less aggressive pathogen than *P. nicotianae*. However, the co-inoculation of both species produced more severe symptoms than inoculation with a single species, indicating an additive effect of these two pathogens and suggesting that opportunistic secondary pathogens like *P. inundata* may have a crucial role in complex diseases.

## 1. Introduction

Citrus, encompassing oranges, lemons, limes, mandarins, pomelos, grapefruits, citrons and kumquats, is one of the most important fruit crop groups worldwide, with cultivation spanning over 140 countries and an annual production exceeding 150 million tons [1]. Among citrus producers in the Mediterranean region, Sicily (southern Italy) is renowned for its primacy in the production of blood oranges [2], which are valuable for the fresh fruit market as well as for juice extraction. Citrus orchards are exposed to numerous biotic and abiotic stresses, many of which can lead to substantial yield losses [3,4,5]. Among the most serious threats are diseases caused by soil-borne oomycetes of the genus *Phytophthora*. These pathogens induce a broad spectrum of symptoms, including root and foot rot, trunk and branch gummosis, canopy blight and fruit brown rot [6,7,8,9]. Gummosis and root rot are particularly severe, often leading to rapid death in young citrus trees, whereas in mature trees the disease typically progresses more slowly and chronically [10]. In almost all major citrus producing countries, the use of tolerant rootstocks is the most popular and effective management option to prevent Phytophthora root and foot rot as well as trunk gummosis [10,11,12,13]. The genus *Phytophthora* encompasses over 250 formally described species, known for their ecological plasticity and broad host range, spanning from agricultural crops to forest ecosystems [14,15,16,17,18]. Around 15 species of *Phytophthora* have been reported to infect or to be associated with citrus [17,19,20,21,22,23,24,25,26,27]. In the Mediterranean region, the most common species causing diseases in citrus orchards are *Phytophthora nicotianae* and *P. citrophthora* [4,25].

Our study focused on a commercial citrus orchard in eastern Sicily, where several mature trees exhibited symptoms of decline, including leaf chlorosis starting from the main veins and expanding to the entire leaf blade, defoliation and root rot, indicative of a possible soil-borne *Phytophthora* infection. To determine the causal agents, soil and fine root samples were collected from symptomatic trees. During this investigation, a *Phytophthora* species with a petaloid colony pattern on potato dextrose agar (PDA), a maximum in vitro growth temperature of 30 °C, and non-papillate sporangia as its most distinctive phenotypic traits was consistently isolated. This species was found in association with *P. nicotianae*, suggesting a potential role in citrus decline.

The objectives of this study were as follows: (I) to identify this *Phytophthora* species previously unreported on citrus in Sicily; (II) to evaluate its pathogenicity on citrus; and (III) to determine its involvement in etiology of root rot and tree decline.

## 2. Results

### 2.1. Isolation and Identification of the Phytophthora Species

The *Phytophthora* species, new for citrus in Sicily, was recovered in a commercial citrus orchard from the rhizosphere of five declining mature trees with symptoms of root rot and decline. It was isolated from soil collected under the tree canopy using leaf baits and directly from fine roots by plating root segments on PARPNH agar medium. Of a total of 42 *Phytophthora* isolates recovered from these five symptomatic trees, 13 were of this species while 39 were tentatively identified as *P. nicotianae* on the basis of the morphological characteristics. The two species showed clearly discernible morphotypes. The isolates of the unidentified species formed petaloid colonies on PDA and colonies without an obvious pattern on V8 agar. Sporangia were non-papillate and persistent, ovoid or obpyriform (30–68 µm in length and 20–52 µm in width, n = 50), sometimes with tapered bases and internal proliferation. They originated from unbranched or simple sympodial sporangiophores, which occasionally displayed swellings. Hyphal swellings were globose, subglobose, irregular, and either individual or catenulated. Chlamydospores were absent. None of the isolates exhibited sexual structures in single culture. Two subsets, one of five isolates of this unidentified species and the other of eight putative isolates of *P. nicotianae*, respectively, were randomly selected for phylogenetic analysis of ITS-rDNA, β-tubulin, and COI sequences. The sequences of isolates of *Phytophthora* species from phylogenetic Clades 1, 4, 6 and 7 were used as references, whereas a *Phytopythium vexans* isolate served as the outgroup (Table 1).

**Figure 1:**
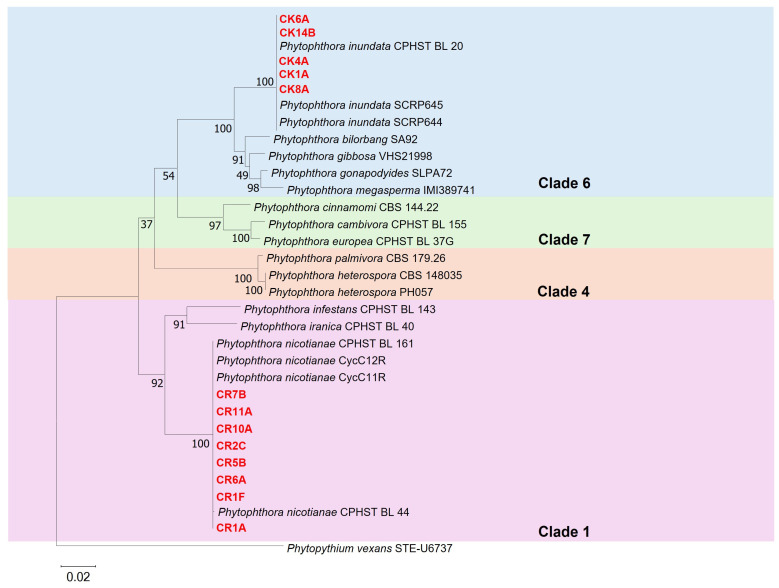
Multilocus phylogenetic tree based on internal transcribed spacer (ITS), β-tubulin (β-tub) and cytochrome c oxidase subunit 1 (COI) sequences, developed using the maximum likelihood method, in accordance with the Tamura–Nei model. The tree with the greatest log likelihood (−4792.34) is shown. Relationships between the 13 *Phytophthora* isolates characterized in this study (highlighted in red), the *Phytophthora nicotianae* and *P. inundata* isolates and other isolates of *Phytophthora* species from Clades 1, 4, 6 and 7 were used as references. *Phytopythium vexans* (isolate STE-U6737) was used as the outgroup.

Isolates identified tentatively as *P. nicotianae* were grouped within Clade 1 along with the reference isolates of *P. nicotianae* (CPHST BL 44, CycC11R, CycC12R and CPHST BL 161), confirming their identity. Conversely, the other five isolates were grouped in Clade 6, together with the reference isolates of *P. inundata* (CPHST BL 20, SCRP645 and SCRP644), and were assigned to this species (Figure 1). The sequences of all isolates characterized in this study were deposited in GenBank (Table 2).

### 2.2. Radial Growth of Phytophthora *spp.* at Different Temperatures

**Figure 2:**
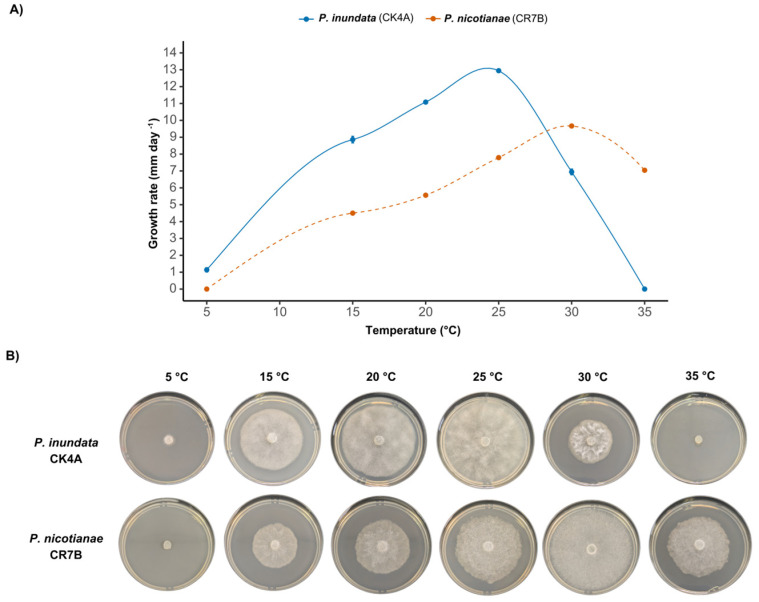
Temperature-dependent radial growth of *Phytophthora inundata* (CK4A) and *Phytophthora nicotianae* (CR7B). (**A**) Growth rate (mm day^−1^) of *P. inundata* and *P. nicotianae* on V8A, measured at six different temperatures, 5, 15, 20, 25, 30 and 35 °C, after 6 and 8 days’ incubation in the dark, respectively. Data points represent mean values and error bars indicate standard deviations. (**B**) Representative colony morphology of *P. inundata* and *P. nicotianae* grown on V8A plates at different temperatures after an incubation period of 6 and 8 days, respectively.

The radial growth rates of *P. inundata* (CK4A) and *P. nicotianae* (CR7B) were evaluated at six different temperatures: 5, 15, 20, 25, 30 and 35 °C (Figure 2). Growth rate measurements, expressed in mm/day, were calculated as the mean radial growth along two perpendicular axes.

Both species exhibited distinct temperature-dependent growth patterns. *P. inundata* reached its maximum growth rate after six days at 25 °C, with an average radial extension of 12.04 ± 0.07 mm/day. In contrast, *P. nicotianae* exhibited its highest growth rate at 30 °C after eight days, with a mean radial extension of 9.66 ± 0.06 mm/day.

At extreme temperatures, differential species responses were observed. At 5 °C, *P. inundata* was able to grow, reaching a radial extension of 1.13 ± 0.13 mm/day after six days, whereas *P. nicotianae* showed no detectable growth. Conversely, at 35 °C, *P. inundata* showed no detectable growth, while *P. nicotianae* was able to grow, reaching a radial extension of 7.04 ± 0.06 mm/day.

### 2.3. Pathogenicity Test

**Figure 3:**
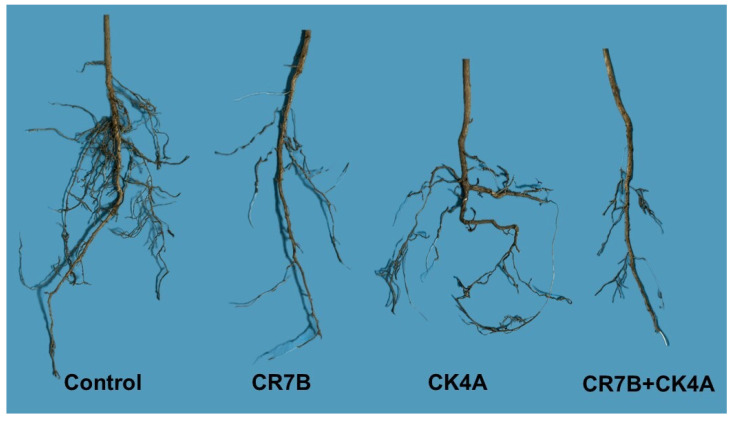
Symptoms of root rot on citrus saplings, 40 days after transplant in soil infested with *P. nicotianae* (CR7B), *Phytophthora inundata* (CK4A) and both *Phytophthora* species (CK4A+CR7B). No symptoms of root rot were observed in control plants.

Since the results of the two separate pathogenicity tests did not differ significantly, as revealed by the analysis of the variance homogeneity, they were pooled and analyzed all together like it was a single experiment. On saplings inoculated with either a single *Phtophthora* species or both species simultaneously, above-ground symptoms at 40 dpi consisted in the chlorosis of main leaf vein and leaf shedding, while control saplings showed no symptoms. No difference was noticed in the severity of above-ground symptoms among saplings inoculated singularly with either *P. inundata* or *P. nicotianae* and those inoculated with both species simultaneously. By contrast, the severity of root damage and the fine root/main root weight ratio varied significantly among the different treatments (Figure 3). Saplings inoculated with *P. nicotianae* (isolate CR7B) showed a severe rot of primary roots and a rarefaction of secondary roots. *P. inundata* (isolate CK4A) was less aggressive. Saplings inoculated with this species showed a denser root apparatus than saplings inoculated with *P. nicotianae* (CR7B) but were sparser than the control saplings. The most severe symptoms of root rot were observed in saplings inoculated with both *P. inundata* and *P. nicotianae* (CR7B+CK4A).

**Figure 4:**
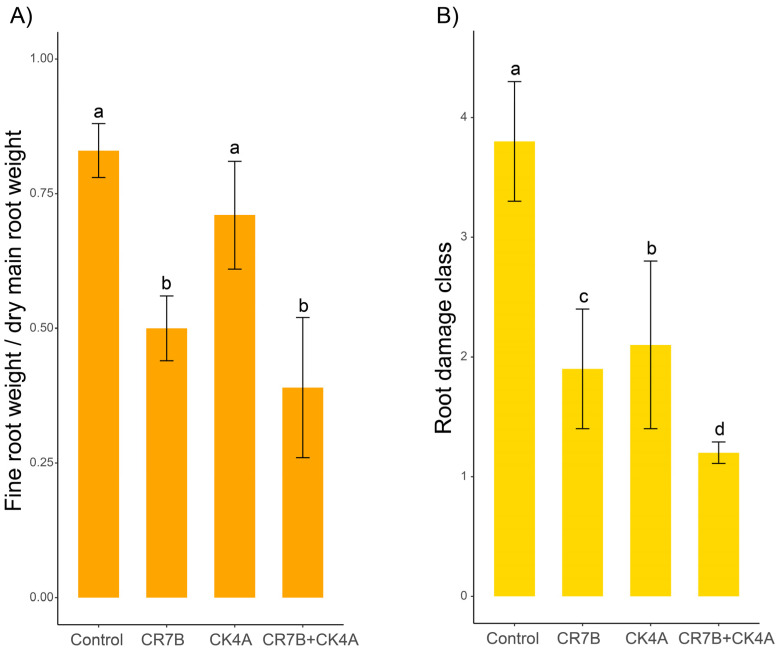
(**A**) The mean fine root/main root weight ratio of saplings of ‘Tarocco Lempso‘ sweet orange on Carrizo’ citrange rootstock, 40 months after transplanting into infested soil, with non-infested soil as control, soil infested with *P. nicotianae* (CR7B), soil infested with *P. inundata* (CK4A) and soil infested with both *Phytophthora* species (CR7B+CK4A). Bars represent standard deviations. Values sharing a common letter are not statistically different according to Tukey’s honestly significant difference (HSD) test (*p* ≤ 0.05). (**B**) The mean root damage class of ‘Tarocco Lempso’ saplings 40 months after transplanting into infested soil, with non-infested soil as control, soil infested with *P. nicotianae* (CR7B), soil infested with *P. inundata* (CK4A) and soil infested with both *Phytophthora* species (CR7B+CK4A). Bars represent standard deviations. Values sharing a common letter are not statistically different according to Tukey’s honestly significant difference (HSD) test (*p* ≤ 0.05).

In saplings inoculated with *P. inundata,* the mean ratio between fine root and main root weight was lower than in control saplings (0.71 ± 0.1) (Figure 4A). However, the difference was not significant, indicating *P. inundata* was weakly pathogenic. By contrast, mean values of the ratio between fine root and main root weight in saplings inoculated with *P. nicotianae*, alone (0.50 ± 0.06) and together with *P. inundata* (0.39 ± 0.13), were significantly lower than in control saplings, indicating a marked detrimental effect on root apparatus. However, the difference between saplings inoculated with *P. nicotianae* alone and with both *P. nicotianae* and *P. inundata* was not significant. As for the root damage class, a similar, albeit more marked, trend was observed (Figure 4B). The mean root damage class of saplings inoculated with *P. inundata* (2.1 ± 0.7) was significantly lower than the control (3.8 ± 0.5). In turn, the mean root damage class of saplings inoculated with *P. nicotianae* (1.9 ± 0.5) was even lower. However, the lowest value of mean root damage class was observed in saplings co-inoculated with *P. nicotianae* and *P, inundata* (1.2 ± 0.09), suggesting an additive effect between these two pathogens. Differences between mean values of root damage class were significant for *p* ≤ 0.05.

Overall, symptoms on inoculated saplings were consistent with those observed in the commercial citrus orchard from which isolates of *P. inundata* and *P. nicotianae* used in pathogenicity tests had been recovered. Both *Phytophthora* species were successfully re-isolated from necrotic roots of inoculated saplings, thereby fulfilling Koch’s postulates. No *Phytophthora* species were obtained from the roots of asymptomatic control seedlings.

## 3. Discussion

*Phytophthora inundata* is a species in *Phytophthora* phylogenetic subclade 6a and, like other species in this clade, it is a common inhabitant of riparian habitats in Europe. Since its formal description [35], this *Phytophthora* species has also been reported from Northern and Southern America, Asia and Australia on various hosts and diverse environments [16,31,36,37,38,39,40]. Recently, Nawza et al. [41] documented a case of crown rot on olive in Pakistan, confirming the ability of this oomycete to infect this host under favorable environmental conditions. In several regions of Italy, including Calabria, Friuli-Venezia Giulia, Sicily, Sardinia and Veneto, *P. inundata* has been recorded in soil of forest ecosystems and as an occasional pathogen of horticultural and ornamental crops, such as olive, peach, walnut and dwarf banana, almost exclusively in flooded or waterlogged soils and mostly associated with other more aggressive *Phytophthora* species [35,42,43,44,45]. Overall, these reports would suggest that *P. inundata* can be regarded as an opportunistic plant pathogen with a broad host range. Moreover, they indicate that this *Phytophthora* species has a wide distribution in Italy. By contrast, in Korea, *P. inundata* has not yet been reported and is regarded as a potentially invasive species; therefore, the possibility of including it in the list of quarantine pathogens is being considered [46]. This study is the first report of *P. inundata* associated with citrus root rot in Europe and the Mediterranean region. Previously, the only documented cases of *P. inundata* affecting citrus were from Chile and India [21,23]. In India, *P. inundata* was identified as the *Phytophthora* species responsible for a decline of Kinnow mandarin trees on rough lemon (*Citrus jambhiri*) as rootstock in waterlogged soils [23]. In Chile, *P. inundata* was recovered from citrus trees affected by root and foot rot in association with *P. citrophthora* [21] but was considered a secondary pathogen; in pathogenicity tests, it proved to be weakly pathogenic which was different to *P. citrophthora*. Both reports are consistent with the findings of the present study. The former highlights that soil waterlogging and root asphyxiation are conducive to infections by *P. inundata*, while the latter suggests that this species, although a weak pathogen on its own, when it is present in multiple infections, may contribute to the decline of citrus trees incited primarily by more aggressive pathogens such as *P. citrophthora* and *P. nicotianae*. As a matter of fact, in the present study on citrus saplings inoculated with both *P. inundata* and *P. nicotianae*, symptoms of root rot were more severe than on saplings inoculated only with the latter species, indicating an additive detrimental effect of the two oomycetes. Although in this study the optimum and high upper limit of temperature for the mycelium growth of *P. inundata* (25 °C and less than 35 °C, respectively) were lower than the optimum and high upper limit of temperature reported in the original description of the species (28–30 °C and around 35–37 °C, respectively) [35], it can be assumed that both *P. inundata* and *P. nicotianae* are mesophilic and prefer a warm climate. It is likely that in the Mediterranean region late spring is the most favorable season for the co-infection of fibrous roots of citrus trees by these two species. In recent decades, the study of complex interactions between plants and multiple microorganisms and the mechanisms by which pathogens interact with each other and affect the phenotypic and genetic response of plants have been gaining special attention [47,48]. The synergistic or additive effect of diverse pathogens and their interactions with both the plant and environmental conditions are crucial to understand the etiology and mechanisms of pathogenesis of complex diseases, such as Phytophthora root rot (PRR) and Dry root rot (DRR), another complex soil-borne disease of citrus trees supposed to be caused by *Fusarium* spp. [5]. PRR and DRR in citrus have some traits in common. Diverse species of *Phytophthora* may be responsible for PRR, as in the case documented in this study, and several *Fusarium* and *Fusarium*-like species differing in virulence are associated with DRR [49]. In both diseases, soil asphyxiation is crucial as predisposing factor and consequently both diseases are particularly damaging in poorly drained or flooded soils. Infections of multiple *Phytophthora* species differing in lifestyle and ecology may have implications for the management of PRR, as even opportunistic pathogens, like *P. inundata*, might impair the resilience of commercial citrus rootstocks in heavy soil, which are more prone to waterlogging. In a recent study of the genetic response of Monterey pine (*Pinus radiata*) seedlings that were inoculated simultaneously with *Fusarium circinatum*, the causal agent of pine pitch canker disease, and the oomycetes *P.* × *cambivora* and *P. parvispora*, it was hypothesized that co-infection might repress the expression of defense-related genes, thus exacerbating the severity of the disease [48]. It is noteworthy that in the Mediterranean region, as a consequence of climate change, during recent years, the frequency of soil water saturation due to more intense precipitations has been increasing. Moreover, warmer temperatures might expand the period of the year favorable to multiple infections of diverse mesophilic *Phytophthora* species associated with the citrus tree rhizosphere.

## 4. Material and Methods

### 4.1. Isolation and Morphological Identification of Isolates

At the end of May 2024, in a 20-year-old commercial orchard in the province of Siracusa, eastern Sicily (geographic coordinates: DATUM WGS 84: 37°18′51.0″ N, 15°02′21.0″ E), soil and fine root samples were collected from five declining mature ‘Tarocco Lempso’ citrus trees grafted on citrange ‘Carrizo’ (*Citrus sinensis* ‘Washington Navel’ × *Poncirus trifoliata*) as rootstock, showing symptoms of root rot and leaf chlorosis starting from the main veins, which are suggestive of Phytophthora root rot. During the same month, meteorological data recorded by the Sicilian Agrometeorological Information Service (SIAS, http://www.sias.regione.sicilia.it/, accessed on 15 December 2024) at the Lentini station indicated a mean daily minimum air temperature of 14.6 °C, a mean daily maximum temperature of 27.7 °C, and a total monthly precipitation of 47.6 mm.

The soil in the orchard was characterized by a clay loam texture and in 2018 had been subjected to flooding. Isolation was performed from the soil using leaf baits in accordance with the protocol described by Riolo et al. [14] as well as directly from fine roots by plating root segments (3–5 mm) on PARPNH agar medium in Petri dishes (9 cm in diameter) and incubating at 22 ± 2 °C in the dark. Subsamples of approximately 400 mL of soil were tested by leaf baiting in a walk-in growth chamber with 12 h of natural daylight at 20 °C. Young leaves of carob (*Ceratonia siliqua*) and oak (*Quercus* spp.) were used as baits. Necrotic pieces (2 × 2 mm) from symptomatic leaves were plated onto selective PARPNH agar medium. Petri dishes were incubated at 22 ± 2 °C, in the dark. Outgrowing hyphae from leaf pieces or root segments were transferred onto V8-juice agar (V8A) under a stereomicroscope. Purified cultures were finally obtained by single hyphal culture on V8-agar. Colony morphology and morphological features of isolates, including the morphology and dimensions of reproductive structures, were determined on colonies grown on V8A at 22 ± 2 °C in the dark according to standard procedures [19]. Sporangia production was stimulated following the method described by Jung et al. [50]. Small fragments (size 2 mm) were cut from the growing edge of 5- to 7-day-old cultures grown in Petri dishes on V8A at 20 °C in the dark. Fragments were placed in 5 cm diameter Petri dishes and flooded with non-sterile soil extract water (prepared by 200 g soil suspended in 1 L of de-ionized water for 24 h at room temperature and then filtered). After incubation at 20 °C in the dark for 24–72 h, dimensions and morphological features of 50 mature sporangia of each isolate were determined at ×400 magnification. All isolates were maintained on V8A and stored at 6 °C in the dark. Other *Phytophthora* isolates preserved in the Molecular Plant Pathology Laboratory collection at the University of Catania were included in this study for comparison.

### 4.2. Assessment of the Effect of Temperature-On Mycelial Growth

The effect of temperature on mycelial growth was evaluated following the methodology described by Scanu et al. [34] with slight modifications. Agar plugs (5 mm in diameter) were excised from actively growing cultures and centrally placed in 90 mm Petri dishes containing V8 agar. Each isolate was incubated in triplicate at temperatures of 5, 15, 20, 25, 30 and 35 °C in the dark.

Radial mycelial growth was monitored daily. Once colonies reached their maximum expansion at the optimal temperature, growth measurements were recorded. To quantify radial growth, two perpendicular lines were drawn on the underside of each Petri dish, intersecting at the center of the inoculum. The radial growth of the colony was recorded along both axes, excluding the initial 5 mm agar plug. The average of these two measurements was then used to determine the growth rate.

### 4.3. Molecular Identification

DNA-based identification of 13 *Phytophthora* isolates that are representative of the two distinct morphotypes obtained from citrus trees in Sicily and randomly selected on the basis of colony morphology (Table 1) was performed via the sequence analysis of the internal transcribed spacer region of ribosomal DNA (ITS-rDNA), β-tubulin (*β-tub*) and cytochrome c oxidase subunit 1 (COI). DNA was extracted from 7-day-old cultures grown on V8A at 20 °C using the PowerPlant Pro DNA Isolation Kit (MO BIO Laboratories, Inc., Carlsbad, CA, USA), following the manufacturer’s instructions. ITS-rDNA was amplified using the ITS6 and ITS4 primers [29]. All PCR reactions were performed in a 25 µL reaction mix containing PCR buffer (1×), dNTP mix (0.2 mM), MgCl2 (1.5 mM), forward and reverse primers (0.5 mM each), Taq DNA Polymerase (1 U) and 100 ng of template DNA. The amplification conditions were as follows: initial denaturation at 94 °C for 3 min, followed by 35 cycles of 94 °C for 30 s, 55 °C for 30 s and 72 °C for 30 s, with a final extension at 72 °C for 10 min [51]. The amplification of β-tubulin and COI regions was performed using the primer pairs TUBUF2/TUBUR1 and COXF4N/COXR4N, respectively [29,52]. Thermocycling conditions included an initial denaturation at 94 °C for 2 min, followed by 35 cycles of 94 °C for 30 s, annealing for 30 s and extension at 72 °C for 60 s, with a final extension at 72 °C for 10 min. Annealing temperatures were set at 60 °C for β-tubulin and 52 °C for COI. Amplicons were visualized on 1% agarose gel and sequenced bidirectionally using an external sequencing service (Macrogen, Amsterdam, The Netherlands). Chromatograms were verified using FinchTV v.1.4.0 [53]. Consensus sequences were initially compared using the BLAST program (https://blast.ncbi.nlm.nih.gov/Blast.cgi, accessed on 4 July 2024) against gene sequences in the NCBI databases and further compared with those of ex-type and other authenticated *Phytophthora* specimens. Phylogenetic analysis was performed using sequences from the isolates in this study alongside validated reference sequences from GenBank (Table 1). Prior to analysis, duplicate reference sequences were removed using Elim Dupes software (https://www.hiv.lanl.gov/content/sequence/elimdupesv2/elimdupes.html, accessed on 4 July 2024)). Sequence alignment was conducted with MUSCLE, and phylogenetic trees, using a combined dataset of all sequenced markers (ITS, β-tubulin and COI), were generated using MEGA 11 [54] with the maximum-likelihood method with the Tamura–Nei model. Bootstrap analysis with 1000 replications was conducted. 

### 4.4. Pathogenicity Tests

The pathogenicity of *Phytophthora* isolates was evaluated in greenhouse experiments using the soil infestation method [29,45,55,56]. Two isolates, one of *Phytophthora inundata* (CK4A) and one of *P. nicotianae* (CR7B), were tested. They were randomly selected among the isolates recovered directly from fine roots. Saplings (12 months old) of ‘Tarocco Lempso’ sweet orange on ‘Carrizo’ citrange rootstock were used as test plants. ‘Carrizo’ citrange is regarded as a rootstock tolerant of Phytophthora root rot [57]. The experiment included four treatments: (I) soil infested with *P. nicotianae* alone, (II) soil infested with *P. inundata* alone, (III) soil infested with both *P. nicotianae* and *P. inundata* and (IV) control, with an equal volume of potting soil and non-infested wheat seeds. It was organized in a randomized block with four blocks and five replicate saplings for each treatment, with a total of 20 saplings per block. For the soil infestation assays, the citrus saplings were transplanted into free-draining pots (12 cm in diameter) containing a 1:1 mixture of autoclaved universal potting soil (Cifo Srl, Giorgio di Piano, Bologna, Italy) and inoculum. The inoculum consisted of a 21-day-old culture grown in the dark at 25 °C in 750 mL flasks containing 50 mL of wheat seeds and 50 mL of V8 juice broth. The inoculum was incorporated into the potting mix at a rate of 20 cm^3^ per 1000 cm^3^. For the combined inoculation treatment, equal volumes of inoculum of *P. inundata* and *P. nicotianae* were mixed together before being incorporated into the potting mix at a rate of 20 cm^3^ per 1000 cm^3^. Control saplings were transplanted into pots with the same sterilized potting mixture without inoculum. After transplanting, all saplings were maintained in saturated soil for 48 h and then transferred to a growth chamber set at 23 °C, with an 80% relative humidity and a photoperiod of 16 h light and 8 h dark. Symptoms were assessed visually at 40 dpi. The effect of inoculation on the above-ground part of saplings was evaluated on the basis of the type and severity of symptoms, while the effect on the roots was evaluated using a root damage scale [56], which allowed a more precise measurement of the virulence of the *Phytophthora* species tested. The root damage scale was as follows: a score of 4 indicated a healthy root system with a dense fine root system and well-developed taproots; a score of 3 indicated less than 25% fine root loss with well-developed taproots; a score of 2 indicated 26–50% fine root loss, initial taproot decay and small necrotic lesions on woody roots or the collar; a score of 1 indicated 51–75% fine root loss, advanced taproot decay and large necrotic lesions on taproots and/or the collar; and a score of 0 indicated 76–100% fine root loss, extensive taproot decay and girdling necrotic lesions on taproots and/or the collar. Moreover, the roots were collected, dried for 72 h at 65 °C and weighed. The ratio between the dry weights of fine roots (diameter < 2 mm) and main roots (diameter 2–10 mm) was recorded for each sapling. Re-isolations of *Phytophthora* from necrotic fresh roots were performed using the selective PARPNH agar medium [56] and the identity of isolates recovered from artificially inoculated symptomatic saplings was confirmed on the basis of morphological characters and molecular analyses. The pathogenicity test was repeated once in the same experimental conditions and with the same experimental design.

### 4.5. Statistical Analysis

The normality of data relative to the effect of inoculation on the sapling roots was preliminarily assessed using the Shapiro–Wilk test while the homogeneity of variance of data from the two separate pathogenicity tests was assessed using the Levene’s test at *p* < 0.05. Then, one-way ANOVA was applied and the differences of means were evaluated with Tukey’s HSD post hoc test for multiple comparisons. Differences were considered statistically significant at *p* < 0.05. All analyses were performed using R software version 4.3.1 (https://www.R-project.org/, accessed on 9 January 2025).

## 5. Conclusions

*Phytophthora inundata* is reported as a pathogen of citrus trees for the first time in Europe and the Mediterranean region. To determine whether this is a one-time occurrence or, by contrast, *P. inundata* is a potential threat for the citrus industry, a more extended survey should be carried out in commercial citrus orchards across Sicily and other citrus producing areas of the Mediterranean macroregion. Preliminary evidence from this study and data from the literature would suggest *P. inundata*, differently from other invasive species such as *P. citrophthora* and *P. nicotianae*, is a weak opportunistic pathogen that favors water-saturated soil. An interesting finding of this study is the additive effect of *P. inundata* infection on the root decay of citrus tree when this species is associated with a more aggressive pathogen, such as *P. nicotianae*. The co-infection of citrus with two oomycetes differing in virulence, such as *P. inundata* and *P. nicotianae*, could be a model system to investigate the complex interactions among host plant and diverse pathogens acting simultaneously and could elucidate the plant defense mechanisms in multiple infections.

## Figures and Tables

**Table 1:** GenBank accession numbers of sequences of the *Phytophthora* spp. isolates used as references and *Phytopythium vexans* used as the outgroup in phylogenetic analyses.

Species	Isolate Code	Clade	Genbank Accession No.			Reference
			ITS	COI	β-Tub	
*Phytophthora iranica*	CPHST BL40	1b	MG865519	MH136913	MH493961	[28]
*P. infestans*	CPHST BL 143	1c	MG865513	MH136907	MH493955	[28]
*P. nicotianae*	CPHST BL 44	1d	MG865550	MH136943	MH493985	[28]
*P. nicotianae*	CPHST BL 161	1d	MG865551	MH477751	MH493987	[28]
*P. nicotianae*	CycC12R	1d	OP557998	OP629938	OP563139	[29]
*P. nicotianae*	CycC11R	1d	OP557997 OP563138	OP629937	OP629937	[29]
*P. heterospora*	CBS 148035	4	MT232394	MZ782830	MZ782809	[30]
*P. heterospora*	PH057	4	MZ927097	MZ782829	MZ782808	[30]
*P. palmivora*	CBS 179.26	4	MT232400	MZ782817	MZ782838	[30]
*P. inundata*	SCRP645	6a	EF210201	EF210207	EF210203	[31]
*P. inundata*	SCRP644	6a	EF210200	EF210206	EF210202	[31]
*P. inundata*	CPHST BL 20	6a	MG865516	MH136910	MH493958	[16]
*P. gibbosa*	VHS21998	6b	HQ012933	HQ012846	JN547596	[32]
*P. gonapodydes*	SLPA72	6b	HQ012937	HQ012850	JN547598	[32]
*P. megasperma*	IMI389741	6b	AF266794	JN935959	JN935977	[33]
*P. bilorbang*	SA92	6c	JN547621	JN547643	JN547582	[32]
*P. cambivora*	CPHST BL155	7a	MG783387	MH136860	MN207270	[28]
*P. europaea*	CPHST BL37G	7a	MG865488	MH136884	MH493935	[28]
*P. cinnamomi*	CBS 144.22	7c	KC478663	KC609419	KC609408	[34]
*Phytopythium vexans*	STE-U6737	-	GU133616	GU133509	GU133454	[28]

**Table 2:** *Phytophthora* spp. isolates obtained from the rhizosphere of declining citrus trees (‘Tarocco Lempso’ grafted on citrange ‘Carrizo’) sampled in a commercial orchard in eastern Sicily. For each isolate, species, isolate code, phylogenetic clade, and GenBank accession numbers for internal transcribed spacer (ITS), cytochrome c oxidase subunit I (COI), and β-tubulin (β-Tub) gene sequences are reported.

Species	Isolate Code	Clade	Genbank Accession No.		
			ITS	COI	β-Tub
*Phytophthora nicotianae*	CR1A	1b	PQ838813	PQ855579	PQ855571
*P. nicotianae*	CR1F	1b	PQ838814	PQ855580	PQ855572
*P. nicotianae*	CR6A	1b	PQ838815	PQ855581	PQ855573
*P. nicotianae*	CR5B	1b	PQ838816	PQ855582	PQ855574
*P. nicotianae*	CR2C	1b	PQ838817	PQ855583	PQ855575
*P. nicotianae*	CR10A	1b	PQ838818	PQ855584	PQ855576
*P. nicotianae*	CR11A	1b	PQ838819	PQ855585	PQ855577
*P. nicotianae*	CR7B	1b	PQ838820	PQ855586	PQ855578
*Phytophthora inundata*	CK1A	6a	PQ838648	PQ855587	PQ855566
*P. inundata*	CK4A	6a	PQ838649	PQ855588	PQ855567
*P. inundata*	CK6A	6a	PQ838650	PQ855589	PQ855568
*P. inundata*	CK8A	6a	PQ838651	PQ855590	PQ855569
*P. inundata*	CK14B	6a	PQ838652	PQ855591	PQ855570

## Data Availability

Data are contained within the article.
